# Immune–inflammatory vulnerability index for risk stratification in older adults with acute exacerbations of COPD: a prospective cohort study

**DOI:** 10.1186/s12877-026-07863-3

**Published:** 2026-06-29

**Authors:** Tingting Huang, Runfeng Sun, Zhaodong Sun, Ming Hu, Xi Jiang, Jiaping Wang, Huiyi Wu, Bo Liu

**Affiliations:** 1 Department of Clinical Laboratory, Donghai Hospital Affiliated to Kangda College of Nanjing Medical University, Donghai County People’s Hospital, Jiangsu, China; 2 Department of Critical Care Medicine, Donghai Hospital Affiliated to Kangda College of Nanjing Medical University, Jiangsu, China; 3 Department of Clinical Laboratory, The First Affiliated Hospital of Kangda College of Nanjing Medical University, The First People’s Hospital of Lianyungang, Jiangsu, China; 4 Central Laboratory, Donghai Hospital Affiliated to Kangda College of Nanjing Medical University, Jiangsu, China

**Keywords:** Acute exacerbation of chronic obstructive pulmonary disease, AECOPD, Immune–inflammatory vulnerability, Immune–Inflammatory Vulnerability Index, PD-1, IL-6, T-cell exhaustion, Risk stratification

## Abstract

**Background:**

Acute exacerbations of chronic obstructive pulmonary disease (AECOPD) are a major cause of morbidity, hospitalization, and short-term mortality in older adults. Growing evidence suggests that aging-related immune remodeling and immune–inflammatory vulnerability (IMV) substantially contribute to biological heterogeneity, susceptibility, and adverse outcomes in this population. However, clinically applicable tools that quantitatively capture this multidimensional biological vulnerability remain limited. This study aimed to develop and externally validate an Immune–Inflammatory Vulnerability Index, hereafter referred to as IAS, conceptualized as a biologically informed index of IMV, to support immune phenotype stratification and short-term risk assessment in older adults hospitalized with AECOPD.

**Methods:**

In this prospective observational cohort study, 219 patients aged ≥ 60 years hospitalized for AECOPD were enrolled in the derivation cohort, and 136 independent patients from an external center were included for validation. Immune phenotyping was performed within 48 h of admission, including PD-1⁺CD4⁺ T cells, CD28⁻CD8⁺ T cells, and serum interleukin-6 (IL-6). IAS was constructed using biologically prespecified immune biomarkers with LASSO-based coefficient weighting, using infection-related versus non-infection-related AECOPD as a supervised biological contrast to enhance immune phenotype differentiation rather than as a direct predictive endpoint. Primary objectives were to evaluate IAS for immune phenotype stratification and to assess its associations with predefined short-term clinical outcomes, specifically 30-day readmission and 90-day all-cause mortality.

**Results:**

IAS demonstrated strong immune phenotype stratification performance for distinguishing infection-related from non-infection-related AECOPD, with an area under the curve (AUC) of 0.842 (95% CI: 0.771–0.901) in the derivation cohort and 0.826 (95% CI: 0.754–0.885) in the external validation cohort. For prediction of 90-day all-cause mortality, IAS achieved an AUC of 0.824 (95% CI: 0.750–0.890). Higher IAS was independently associated with increased 30-day readmission, prolonged hospitalization, and elevated 90-day mortality risk after adjustment for age, disease severity, and established clinical risk scores (adjusted HR = 3.42, 95% CI: 2.01–5.82). Sensitivity analyses showed that IAS outperformed IL-6 alone and retained discriminatory capacity even after exclusion of IL-6, supporting its broader immune–inflammatory relevance beyond acute inflammatory burden. Longitudinal analyses demonstrated partial remission-associated declines in IAS, consistent with state-responsive biological vulnerability rather than a fixed immune-aging trait.

**Conclusions:**

IAS is a biologically informed, geriatric-oriented index of IMV that integrates aging-related immune remodeling with dynamic inflammatory responses in older adults with AECOPD. Rather than serving as a definitive measure of fixed immune-aging burden or as a dedicated infection classification tool, IAS provides a quantitative framework for biologically relevant immune–inflammatory vulnerability stratification and short-term prognostic assessment. Further prospective multicenter studies are warranted to refine clinical applicability, evaluate implementation feasibility, and improve translational potential before routine clinical adoption can be recommended.

**Supplementary Information:**

The online version contains supplementary material available at 10.1186/s12877-026-07863-3.

## Introduction

Chronic obstructive pulmonary disease (COPD) is often complicated by acute exacerbations (AECOPD), which accelerate lung function decline and significantly increase hospitalization and mortality rates [1–3]. AECOPD can be triggered by various factors, including bacterial and viral infections, environmental pollution, allergen exposure, metabolic disorders, and poor treatment adherence [4, 5]. In clinical practice, infection-related and non-infection-related exacerbations often present with overlapping symptoms and inflammatory markers, making accurate classification challenging. Increasing evidence suggests that distinct immune response mechanisms may underlie these different triggers [6, 7].

However, current risk stratification approaches in AECOPD are largely based on inflammatory markers, which may not adequately capture immune–inflammatory vulnerability (IMV), particularly in older adults.

Recently, “immune aging” has been recognized as a critical biological factor influencing the progression of chronic diseases and acute exacerbations. Immune aging is characterized by T cell exhaustion (upregulation of PD-1⁺ expression), terminal differentiation of memory T cells (accumulation of CD28⁻ T cells), metabolic reprogramming, and low-level systemic inflammation (e.g., persistently elevated IL-6, TNF-α) [8–10]. In COPD patients, prolonged exposure to oxidative stress and chronic inflammation accelerates immune aging, leading to impaired immune function and dysregulated inflammation control [11, 12]. This process is closely linked to abnormalities in the mTOR/AMPK signaling pathway and mitochondrial dysfunction, which reduce the body’s immune adaptability to external stimuli [13]. Thus, COPD is not only a respiratory disease but also a systemic condition characterized by immune homeostasis imbalance [14, 15].

Importantly, COPD is increasingly recognized as a heterogeneous disease with diverse physiological, molecular, and clinical subtypes. Large cohort studies and multidimensional phenotyping frameworks have demonstrated substantial heterogeneity in inflammatory pathways, transcriptomic signatures, imaging characteristics, and prognosis among COPD populations. Landmark observational cohorts, including COPDGene, SPIROMICS, and ECLIPSE, have further shown that COPD populations differ substantially in clinical presentation, exacerbation susceptibility, imaging phenotypes, disease progression, and underlying biological pathways [16–18]. These cohorts have established phenotype-driven approaches as an important foundation for risk stratification and precision medicine in COPD. Recent evidence from Fangal et al. further demonstrated that obstructive airway diseases, including COPD and asthma–COPD overlap, exhibit distinct yet partially overlapping physiological and transcriptomic endotypes, reinforcing the need for biologically informed phenotyping beyond traditional symptom-based or trigger-based classification [19, 20]. However, immune-related heterogeneity during acute exacerbations, particularly in older adults, remains insufficiently characterized. These findings suggest that aging-related immune remodeling may represent an underrecognized axis of heterogeneity in older AECOPD, particularly when differentiating infection-triggered from non-infection-triggered exacerbations.

Different immune aging profiles may profoundly affect the mechanisms behind AECOPD and its clinical outcomes. Previous studies have found that an increased proportion of PD-1⁺ T cells correlates with excessive responses to endogenous danger-associated molecular patterns (DAMPs), amplifying systemic inflammation. Conversely, the accumulation of CD28⁻ T cells suggests immune metabolic remodeling, maintaining low-grade inflammation even without clear infection triggers [11–13, 21]. Recently, the “comparatively lower inflammatory burden with persistent immune senescence-related vulnerability” immune phenotype has been identified in older adults with non-infection-related AECOPD and is associated with recurrent exacerbations and poor prognosis [22]. In this state, traditional inflammatory markers (e.g., CRP, IL-6) may not show significant elevation, but immune response capacity is compromised, making patients more vulnerable to metabolic disturbances, environmental stressors, and comorbidities [12, 15, 23].

These findings highlight that inflammation-based indicators alone may be insufficient to fully characterize the biological heterogeneity of AECOPD, particularly in aging populations.

Despite the growing recognition of immune aging’s role in AECOPD, there is still no widely applicable, quantifiable tool for assessing its clinical phenotype. Furthermore, the differential mechanisms of immune aging in infection-related and non-infection-related AECOPD remain insufficiently explored. To address this gap, we developed an Immune–Inflammatory Vulnerability Index, hereafter referred to as IAS, based on PD-1⁺CD4⁺ T cells, CD28⁻CD8⁺ T cells, and IL-6. This score systematically evaluates its utility in classifying infection-related and non-infection-related AECOPD and explores its association with short-term adverse outcomes, specifically 30-day readmission and 90-day all-cause mortality.

Unlike conventional single inflammatory markers, IAS is designed as a composite framework integrating immune senescence, T-cell exhaustion, and systemic inflammation, thereby capturing a broader dimension of immune–inflammatory vulnerability.

This study aimed to develop a quantifiable IAS to characterize IMV and support immune phenotype stratification in older adults hospitalized with AECOPD. Specifically, IAS was designed to distinguish infection-related from non-infection-related AECOPD and to evaluate its associations with predefined short-term outcomes, including 30-day readmission and 90-day all-cause mortality. Rather than representing a direct measure of immune aging, IAS is conceptualized as a biologically informed, geriatric-oriented IMV index that integrates immune remodeling and dynamic inflammatory responses in older adults with AECOPD.

## Materials and methods

### Study design and participants

This prospective observational study included a derivation cohort from Donghai County People’s Hospital and an independent external validation cohort from the First Affiliated Hospital of Kangda College of Nanjing Medical University, between January 2023 and December 2024. All participants met the GOLD 2023 criteria [24] and had confirmed FEV₁/FVC < 0.70 through pulmonary function tests [25, 26].

**Fig. 1:**
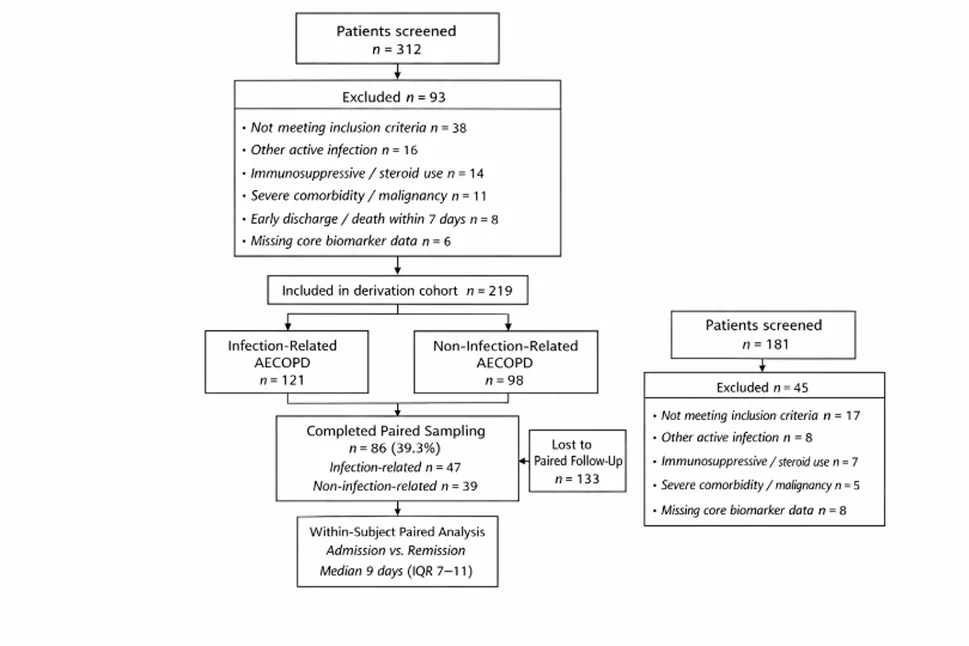
Flow diagram of patient selection, including enrollment, exclusion criteria, and final inclusion in the derivation and external validation cohorts

The study was reported in accordance with the STROBE guidelines, and a completed checklist is provided in Supplementary Table S1. A participant flow diagram is presented in Fig. 1.

An independent external validation cohort was recruited from the First Affiliated Hospital of Kangda College of Nanjing Medical University during the same study period (January 2023 to December 2024), using identical inclusion and exclusion criteria. This design ensured methodological consistency and allowed for independent assessment of the generalizability of the IAS.

Inclusion Criteria: Age ≥ 60 years; admission due to acute exacerbation of COPD (AECOPD), with worsening respiratory symptoms (such as cough, sputum, or dyspnea) requiring adjustment of the treatment plan; completion of C-reactive protein (CRP), procalcitonin (PCT), and immune phenotyping within 48 h of admission.

Exclusion Criteria: Presence of other active infections (e.g., pneumonia, sepsis) with a primary diagnosis other than AECOPD; use of immunosuppressive therapy or systemic corticosteroids (equivalent to prednisone ≥ 10 mg/day) in the past 3 months; severe immune dysfunction diseases (e.g., malignancy, autoimmune diseases, or HIV infection); death or voluntary discharge within 7 days of admission, leading to missing core data.

### Definition and classification of infection-related and non-infection-related AECOPD

Infection-related AECOPD was diagnosed using a multidimensional adjudication framework incorporating clinical, laboratory, microbiological, and radiographic evidence. Patients were classified as having infection-related AECOPD if any of the following criteria were met:

- (1) Positive pathogen detection by conventional microbiological methods or metagenomic next-generation sequencing (mNGS);
- (2) Elevated inflammatory biomarkers (CRP ≥10 mg/L or PCT ≥0.5 ng/mL) accompanied by clinical manifestations consistent with infectious exacerbation.

For patients with discordant microbiological, biomarker, or clinical findings, infection status was independently adjudicated by two senior respiratory physicians using predefined hierarchical criteria, including microbiological evidence, inflammatory biomarkers, clinical symptoms, and radiographic findings. Adjudicators were blinded to immune phenotyping results and IAS values, and disagreements were resolved through consensus discussion. To minimize potential misclassification, no systemic antibiotics were administered within 48 h before sample collection.

The same adjudication framework, including microbiological evidence, CRP/PCT thresholds, clinical manifestations, and specialist review procedures, was applied unchanged in the external validation cohort.

Importantly, CRP and PCT were used solely to assist infection classification and were not included in IAS construction. Infection-related versus non-infection-related AECOPD served only as a supervised biological contrast for immune phenotype differentiation during model development rather than as a predictive endpoint. This strategy was intended to facilitate identification of biologically relevant immune–inflammatory vulnerability patterns while minimizing bias arising from overlapping inflammatory signals.

Non-infection-related AECOPD was generally associated with noninfectious triggers such as airway irritation, environmental exposure, metabolic disturbances, heart failure, or poor treatment adherence. Environmental exposure was defined as documented exposure within 7 days before admission to one or more recognized noninfectious triggers, including ambient air pollution, active or passive tobacco smoke, occupational dust or chemical fumes, biomass fuel smoke, or abrupt temperature changes. Exposure status was determined through structured clinical history obtained at admission.

The diagnosis and classification procedures were developed in accordance with GOLD 2023 and ERS/ATS recommendations. For mNGS-positive cases, interpretation incorporated read counts, relative abundance, background database correction, and negative controls, with potential contaminants assigned lower diagnostic weight.

### Immune phenotyping and biomarker measurement

Clinical data, including demographic characteristics, smoking history, comorbidities, laboratory parameters, and clinical outcomes, were prospectively collected within 48 h of hospital admission. Peripheral venous blood samples were obtained during the acute admission phase for routine laboratory testing and immune phenotyping. Flow cytometry was performed to quantify PD-1⁺CD4⁺ T cells, PD-1⁺CD8⁺ T cells, and CD28⁻CD8⁺ T cells using standardized antibody panels and predefined gating strategies. Serum IL-6 levels were measured using enzyme-linked immunosorbent assay (ELISA) according to manufacturer protocols. mHLA-DR was additionally measured in a subset of patients as an exploratory immune status marker for secondary subgroup analyses but was not included in IAS derivation. IAS construction was intentionally restricted to three biologically prespecified core markers (PD-1⁺CD4⁺, CD28⁻CD8⁺, and IL-6) to preserve model parsimony, reduce overfitting risk, and enhance reproducibility across cohorts.

Immune phenotyping assays in the external validation cohort were performed independently at the First Affiliated Hospital of Nanjing Medical University Kangda College under standardized and comparable laboratory conditions, including consistent antibody panels, flow cytometry platforms, gating strategies, and quality-control procedures.

Remission was defined as clinical stability at discharge, characterized by resolution of acute exacerbation symptoms (including no worsening of cough, dyspnea, purulent sputum, or fever) and stable vital signs. For patients undergoing longitudinal follow-up, remission-phase peripheral blood samples were collected within 24 h prior to hospital discharge using the same standardized immune phenotyping protocol.

Of the 219 patients included in the primary cohort, 83 completed paired admission-remission immune phenotyping and were included in longitudinal analyses. Patients without complete paired sampling were excluded from longitudinal analyses due to incomplete sample collection or insufficient immune cell quantification. Baseline demographic and clinical characteristics between paired completers and non-completers were compared to assess representativeness.

All blood samples were processed within 2 h of collection. Flow cytometry analyses were performed by trained personnel blinded to infection classification using standardized operating procedures to minimize inter-operator variability.

Cardiac biomarkers and cardiovascular functional parameters, including Troponin T, NT-proBNP, and left ventricular ejection fraction (LVEF), were not routinely measured and were therefore not included in the present analysis.

### Construction of the IAS

The IAS, retained as the established abbreviated name of the index, was developed using three prespecified immune-related biomarkers: PD-1⁺CD4⁺ T cells, CD28⁻CD8⁺ T cells, and serum IL-6. These markers reflect T-cell exhaustion, terminal differentiation, and systemic inflammation, which are key components of IMV. Rather than representing a direct measure of immune aging, IAS was designed as a composite index integrating immune remodeling and dynamic inflammatory responses.

The candidate variable set submitted to LASSO consisted exclusively of these three prespecified biomarkers.Therefore, LASSO was used primarily for coefficient shrinkage and weight estimation among biologically prespecified immune markers, rather than broad exploratory variable screening.

Before model construction, all variables were standardized using the Z-score method:

$$\mathrm{Z}\_\mathrm{i}\;=\;(\mathrm{X}\_\mathrm{i}\;-\;\mu\_\mathrm{i})\;/\;\sigma\_\mathrm{i}$$

where X_i is the individual observation, and µ_i and σ_i are the mean and standard deviation of the corresponding variable in the derivation cohort.

LASSO logistic regression was performed with non-infection-related AECOPD coded as the dependent outcome and infection-related AECOPD as the reference category. This biologically informed framework was used to identify biomarkers independently associated with the non-infection-related IMV phenotype after adjustment, rather than to compare raw inflammatory burden between infection-related and non-infection-related groups. Ten-fold cross-validation was applied to determine the optimal penalty parameter (λ_min). Variables with non-zero coefficients were retained, and their coefficients were used as IAS weights.

All three prespecified biomarkers retained non-zero coefficients in the final model. The standardized coefficients were 0.38 for PD-1⁺CD4⁺ T cells, 0.33 for CD28⁻CD8⁺ T cells, and 0.47 for log₁₀(IL-6). The raw IAS was calculated as follows:

$$\begin{aligned}\text{Raw IAS}\;=&\;0.38\;\times\;\mathrm{Z}(\mathrm{PD}-1^+\mathrm{CD4}^+\;\text{T cells})\;\\&+\;0.33\;\times\;\mathrm{Z}(\mathrm{CD28}^-\mathrm{CD8}^+\;\text{T cells})\;\\&+\;0.47\;\times\;\mathrm{Z}(\mathrm{log}_{10}\mathrm{IL}-6)\end{aligned}$$

$$\begin{aligned}\text{Scaled IAS}\;&=\;[(\text{Raw IAS}\;-\;\text{Raw IAS}\_\mathrm{min})\;/\;\\&(\text{Raw IAS}\_\mathrm{max}\;-\;\text{Raw IAS}\_\mathrm{min})]\;\times\;10\end{aligned}$$

The same derivation-cohort scaling parameters were applied unchanged to the external validation cohort. –Unless otherwise specified, references to IAS cutoff values refer to the rescaled 0–10 IAS.–.

For categorical prognostic analyses, the optimal IAS cutoff was prespecified in the derivation cohort using receiver operating characteristic (ROC) analysis for 90-day all-cause mortality, with the Youden index used to maximize sensitivity and specificity. The resulting threshold was rescaled IAS = 5.30 (sensitivity 78.0%; specificity 74.2%). Patients with IAS ≥ 5.30 were classified as high IAS, whereas those with IAS < 5.30 were classified as low IAS. This cutoff was fixed after derivation and applied unchanged to Kaplan–Meier analyses, multivariable prognostic models, subgroup analyses, and the external validation cohort without recalibration.

IAS was also analyzed as a continuous variable in regression models to preserve statistical power and minimize information loss caused by categorical stratification. –In addition to categorical prognostic analyses, IAS was analyzed as a continuous variable in regression models to preserve statistical power and minimize information loss caused by risk stratification using the prespecified ROC-Youden threshold.– A higher rescaled IAS value indicates greater immune–inflammatory vulnerability rather than a pure increase in immune aging burden.

### Model development logic and sensitivity analysis

IAS was designed to characterize immune–inflammatory vulnerability rather than to directly predict clinical outcomes. During IAS derivation, non-infection-related AECOPD was prespecified as the dependent outcome variable, with infection-related AECOPD serving as the reference category. This biologically informed framework was selected to enhance identification of biomarkers independently associated with the non-infection-related immune–inflammatory vulnerability phenotype after multivariable adjustment. Importantly, infection classification served as a supervised biological contrast to facilitate immune phenotype differentiation rather than as a direct clinical endpoint or a simple between-group comparison of raw inflammatory burden. Consequently, IAS should be interpreted as a biologically informed index of immune–inflammatory vulnerability rather than a direct measure of fixed immune-aging burden.

Subsequent analyses evaluated associations between IAS and clinical outcomes separately to assess whether this biologically derived score could capture geriatric vulnerability and prognostic relevance beyond traditional inflammatory markers.

Given the relatively small number of 90-day mortality events, directly modeling mortality as the outcome could introduce outcome-driven bias and reduce model stability. To assess this potential limitation, we performed an alternative LASSO analysis using 90-day mortality as the outcome and compared the results with the IAS model. The mortality-based model demonstrated poorer stability in the external validation cohort (AUC decreased by approximately 0.06–0.09), suggesting limited generalizability. In contrast, the IAS model, which focuses on immune phenotype differences, exhibited superior discrimination and stability in both internal and external cohorts, supporting its validity as a tool for capturing IMV.

Continuous variables were expressed as mean ± standard deviation (SD) for normally distributed data or median (interquartile range [IQR]) for non-normally distributed data, while categorical variables were presented as frequency and percentage. Between-group comparisons were performed using independent-samples t test for normally distributed continuous variables or Mann–Whitney U test for non-normally distributed variables, as appropriate. Paired comparisons were conducted using paired t test or Wilcoxon signed-rank test according to data distribution.

Missingness was low across all study variables. Specifically, missing data rates were 0.9% (2/219) for PD-1⁺CD4⁺ T cells, 1.4% (3/219) for CD28⁻CD8⁺ T cells, 1.8% (4/219) for IL-6, 0.5% (1/219) for CRP, 0.9% (2/219) for PCT, 0.9% (2/219) for GOLD stage, and 1.4% (3/219) for BODE score. No variable exceeded 5% missingness, and no systematic missingness pattern was identified; therefore, complete-case analysis was applied.

LASSO regression was used to select immune-related variables for constructing IAS. Model discrimination ability was assessed using receiver operating characteristic (ROC) curves and area under the curve (AUC), with internal validation performed through 1,000 bootstrap resamples. Separate ROC analyses were conducted for two distinct purposes: (1) immune phenotype classification, distinguishing infection-related from non-infection-related AECOPD; and (2) prognostic prediction of 90-day all-cause mortality. AUC values were interpreted according to their specific analytical endpoint and cohort context. Comparisons between ROC curves of IAS and conventional prognostic models, including BODE, DECAF, and combined models, were performed using the DeLong test. The AUC reported for distinguishing infection-related and non-infection-related AECOPD reflects phenotype discrimination rather than independent validation of an infection classification task. All variable standardization methods, weight coefficients, and model structures were kept consistent when applied to the external validation cohort to ensure reproducibility and generalizability.

Exploratory unsupervised clustering was performed using the k-means algorithm to investigate immune phenotype heterogeneity. PD-1⁺CD4⁺ T cells, CD28⁻CD8⁺ T cells, and IL-6 were selected a priori based on biological relevance and mechanistic contribution to IAS. All variables were standardized using z-score normalization before analysis, and Euclidean distance was applied within the k-means framework. The optimal number of clusters was determined by combining silhouette coefficient analysis with consensus clustering based on 1,000 resampling iterations, supporting a three-cluster solution. The final model achieved a silhouette score of 0.72, indicating good internal consistency. This clustering analysis was exploratory rather than prespecified and was not used for IAS construction, primary endpoint definition, or direct validation of the supervised IAS-derived immune phenotype framework.

A Cox proportional hazards model was used to evaluate the association between IAS and 90-day all-cause mortality, with the proportional hazards assumption assessed using Schoenfeld residuals. In the derivation cohort, 41 patients experienced 90-day all-cause mortality and 34 experienced 30-day readmission. The primary multivariable Cox model included seven predictors, namely IAS, age, sex, GOLD stage, CRP, PCT, and BODE score, yielding an events-per-variable ratio of approximately 5.9 for mortality analyses. Given the relatively limited event number, Firth penalized likelihood estimation was applied to reduce small-sample bias and improve coefficient stability.

To assess the robustness, calibration performance, and potential clinical utility of IAS, supplementary statistical analyses were conducted. Model calibration was evaluated through bootstrap resampling with 1,000 iterations, and bias-corrected calibration curves were constructed. Brier scores and the Hosmer–Lemeshow goodness-of-fit test were used to quantify agreement between predicted probabilities and actual outcomes. Decision curve analysis (DCA) was performed to evaluate the net clinical benefit of IAS across different threshold probability ranges and to compare its performance with traditional risk scoring systems, including BODE and DECAF, in both the derivation and external validation cohorts.

These supplementary analyses were used solely for model performance verification and did not influence model construction or primary endpoint definition. The final IAS was calculated as a weighted linear combination of standardized variables based on the LASSO-derived coefficients, which are provided in Supplementary Table S2.

## Results

### Patient characteristics and grouping

This study included 219 older adults (age ≥ 60 years) hospitalized with acute exacerbations of chronic obstructive pulmonary disease (AECOPD) on a stable COPD baseline. Patients were classified into infection-related AECOPD (*n* = 121) and non-infection-related AECOPD (*n* = 98) groups based on a predefined adjudication framework incorporating clinical features, imaging findings, inflammatory biomarkers (CRP and PCT), and microbiological evidence. During follow-up, 34 of 219 patients (15.5%) experienced 30-day readmission and 41 (18.7%) died within 90 days.

Of the 121 patients classified as infection-related AECOPD, 74 (61.2%) had microbiologically confirmed infection based on positive pathogen detection by sputum culture, blood culture, respiratory viral testing, or metagenomic next-generation sequencing (mNGS). Another 31 patients (25.6%) were classified as clinically probable infection-related AECOPD because they presented with compatible infectious symptoms together with elevated inflammatory biomarkers (CRP ≥ 10 mg/L and/or PCT ≥ 0.5 ng/mL), despite negative or unavailable microbiological results. The remaining 16 patients (13.2%) showed discordant microbiological, biomarker, or clinical findings and therefore underwent independent review by two senior respiratory physicians who were blinded to immune phenotyping results. Any disagreements were resolved through consensus discussion. The initial inter-rater agreement for these uncertain cases was substantial (Cohen’s κ = 0.82). In contrast, all 98 patients classified as non-infection-related AECOPD lacked microbiological confirmation and did not meet predefined biomarker or clinical criteria sufficient for infection-related classification.

During the 90-day follow-up period, a total of 41 patients experienced all-cause mortality, which formed the basis for subsequent survival and prognostic analyses.

There were no statistically significant differences between the two groups in baseline characteristics, including age, sex, smoking history, and major comorbidities such as hypertension and coronary artery disease (all *P* > 0.05). However, the infection-related group had significantly longer hospital stays and higher antibiotic usage rates compared to the non-infection-related group (both *P* < 0.001), as shown in Table 1. At admission, infection-related AECOPD demonstrated higher absolute inflammatory and immune activation marker levels; therefore, IAS should be interpreted as reflecting broader immune–inflammatory vulnerability rather than a simple binary exhaustion phenotype. Table 1 presents baseline immune marker levels measured at admission and reflects raw group comparisons.

**Table 1:** Comparison of General Data and Key Immune–inflammatory Features Between Infection-related and Non-infection-related AECOPD Patients

Variable	Infection-related AECOPD Group (*n* = 121)	Non-infection-related AECOPD Group (*n* = 98)	*P*-value*
Age (years)	73.2 ± 6.5	72.1 ± 5.9	0.421
Male sex, n (%)	84 (69.4%)	66 (67.3%)	0.743
Smoking history, n (%)	70 (58.7%)	56 (57.1%)	0.814
Chronic heart failure comorbidity, n (%)	39(32.5)	34(35.0)	0.793
CRP (mg/L)	98.4 [67.8–115.8]	12.5 [8.7–15.7]	< 0.001
PCT (ng/mL)	8.87 [6.72–10.98]	0.18 [0.11–0.29]	< 0.001
IL-6 (pg/mL)	267.2 [218.1–356.9]	36.4 [24.6–50.9]	< 0.001
CD28⁻CD8⁺ T cells (%)	41.2 ± 10.5	28.9 ± 9.7	< 0.001
PD-1⁺CD4⁺ T cells (%)	19.5 ± 4.6	11.7 ± 3.1	< 0.001
CD4⁺/CD8⁺ ratio	1.47 ± 0.58	1.39 ± 0.54	0.518
NLR (Neutrophil/Lymphocyte ratio)	5.9 ± 2.1	3.3 ± 1.5	< 0.001
Length of hospital stay (days)	11.8 ± 4.2	8.6 ± 3.3	0.002

*P-values were calculated using Student’s t-test for normally distributed continuous variables, Mann–Whitney U test for skewed continuous variables, and χ² test for categorical variables, as appropriate

An independent external validation cohort comprising 136 patients was included, as described in the Methods section. Baseline characteristics between the derivation and validation cohorts were generally comparable (Supplementary Table S3). No significant differences were observed in key demographic variables, inflammatory markers, or immune phenotyping parameters, supporting the comparability and generalizability of the study population.

### Immune phenotype classification performance of IAS for distinguishing infection-related versus non-infection-related AECOPD

IAS demonstrated strong discriminatory ability in distinguishing infection-related from non-infection-related AECOPD. This discrimination reflects the model’s capacity to capture differences in underlying immune phenotypes and IMV rather than serving as a diagnostic classifier for infection.

In the derivation cohort, IAS demonstrated strong immune phenotype classification performance for distinguishing infection-related versus non-infection-related AECOPD (AUC = 0.842, 95% CI: 0.771–0.901). This classification performance remained stable in the independent external validation cohort (AUC = 0.826, 95% CI: 0.754–0.885). Applying the same predefined cutoff (Rescaled IAS = 5.30) to the external validation cohort classified 58 patients (42.6%) as high IAS and 78 (57.4%) as low IAS. Phenotype-classification performance remained stable without recalibration, supporting the reproducibility and transportability of the IAS framework across independent populations.

It should be noted that this analysis represents phenotype discrimination rather than independent validation of predictive performance. The model’s calibration performance, internal validation results, and decision curve analysis are presented in Supplementary Figures S1–S2. Clinical outcome event distributions for both cohorts are summarized in Supplementary Table S4.

To evaluate whether IAS performance was primarily driven by acute inflammatory signals, sensitivity analyses were conducted. IL-6 alone yielded an AUC of 0.72, which was lower than that of the full IAS (AUC = 0.842). A reduced model excluding IL-6, incorporating PD-1⁺CD4⁺ and CD28⁻CD8⁺ T cells, achieved an AUC of 0.79. These findings indicate that IAS integrates broader immune-related information beyond a single inflammatory biomarker, supporting its role as a composite indicator of IMV rather than a single-pathway inflammatory or static immune aging metric.

#### Sensitivity analyses using stricter infection definitions

To evaluate the robustness of IAS against potential infection misclassification, two sensitivity analyses were conducted. First, when infection-related AECOPD was restricted to microbiology-confirmed cases only (*n* = 74), IAS maintained strong discriminatory performance, with an AUC of 0.831 (95% CI: 0.756–0.892). Second, after excluding all specialist-adjudicated uncertain cases (*n* = 16), the discriminatory ability of IAS remained stable (AUC = 0.838, 95% CI: 0.768–0.896). Across both stricter classification strategies, higher IAS remained significantly associated with prolonged hospitalization, increased 30-day readmission, and elevated 90-day mortality risk, with effect estimates comparable to the primary analysis. These findings support that the biological and prognostic relevance of IAS was not materially driven by uncertain infection classification.

### Differences in immune–inflammatory profiles between infection-related and non-infection-related AECOPD patients

At admission, infection-related and non-infection-related AECOPD patients exhibited distinct immune–inflammatory profiles. The infection-related group demonstrated significantly higher systemic inflammatory burden, particularly elevated IL-6 levels, consistent with acute inflammatory activation driven by infectious triggers. In contrast, baseline immune phenotyping suggested substantial heterogeneity beyond inflammatory intensity alone.

In multivariable-adjusted logistic regression analyses (Table 2), when non-infection-related AECOPD was modeled as the dependent phenotype, higher admission-phase PD-1⁺CD4⁺ T-cell levels (OR 1.46, 95% CI 1.15–1.85) and CD28⁻CD8⁺ T-cell levels (OR 1.39, 95% CI 1.12–1.73) were independently associated with the non-infection-related phenotype, whereas higher log₁₀(IL-6) remained negatively associated (OR 0.63, 95% CI 0.48–0.82), indicating stronger linkage to infection-related AECOPD. These findings suggest that, after adjustment, non-infection-related AECOPD showed features suggestive of a relatively “lower inflammation–higher exhaustion” immune–inflammatory vulnerability pattern in this cohort. Importantly, these adjusted phenotype associations should not be interpreted as direct contradictions to Table 1, which reflects unadjusted raw admission inflammatory burden.

**Table 2:** Multivariable-adjusted cross-sectional associations of admission-phase immune biomarkers with non-infection-related AECOPD phenotype

Variable	β Coefficient	Standard Error	OR (95% CI)	*P*-value
PD-1⁺CD4⁺ T cells (Z-score)	0.38	0.12	1.46 (1.15–1.85)	0.002
CD28⁻CD8⁺ T cells (Z-score)	0.33	0.10	1.39 (1.12–1.73)	0.004
log10(IL-6) (Z-score)	−0.47	0.14	0.63 (0.48–0.82)	< 0.001

This table presents multivariable-adjusted associations from a logistic regression model in which non-infection-related AECOPD was the dependent outcome (reference: infection-related AECOPD). OR > 1 indicates an independent positive association with the non-infection-related group, whereas OR < 1 indicates a stronger association with the infection-related group. These findings reflect adjusted cross-sectional association patterns based on admission-phase biomarker measurements and should not be interpreted as definitive biological phenotypes or causal immune subtypes

These observations indicate differential IMV-associated patterns between infection-related and non-infection-related AECOPD, but should be interpreted cautiously. While infection-related exacerbations were characterized predominantly by acute inflammatory intensity, non-infection-related AECOPD showed adjusted biomarker associations suggestive of stronger T-cell exhaustion and terminal differentiation. Collectively, this pattern may reflect biologically distinct immune–inflammatory profiles across AECOPD subtypes, yet inflammatory burden alone may not fully capture the multidimensional immune vulnerability of older adults with AECOPD.

In the adjusted multivariable model (Table 2), PD-1⁺CD4⁺ T cells and CD28⁻CD8⁺ T cells were positively associated with non-infection-related AECOPD, whereas IL-6 was negatively associated with this phenotype, further supporting a pattern that is suggestive rather than definitive of “lower inflammation–higher exhaustion”IMV in this cohort. Because Table 2 presents multivariable-adjusted associations using non-infection-related AECOPD as the dependent variable and infection-related AECOPD as the reference category, OR > 1 indicates an independent positive association with the non-infection-related phenotype, whereas OR < 1 indicates stronger association with infection-related AECOPD. These adjusted associations reflect phenotype-specific biomarker relationships after covariate adjustment rather than raw baseline biomarker differences shown in Table 1, and should be interpreted cautiously rather than as absolute value comparisons.

### Dynamic changes in IAS during admission and remission

A longitudinal analysis of patients who completed paired admission–remission testing (*n* = 83) revealed significant declines in several immune–inflammatory markers during disease remission. T cell exhaustion markers (PD-1⁺CD4⁺), terminal differentiation markers (CD28⁻CD8⁺), and systemic inflammatory markers (IL-6) all decreased significantly from admission to remission (P-values < 0.001, 0.002, and < 0.001, respectively).

In the overall paired cohort (*n* = 83), significant reductions in IL-6, PD-1⁺CD4⁺, CD28⁻CD8⁺ T cells, and IAS were observed from admission to remission. These findings indicate partial recovery of immune–inflammatory perturbations during clinical stabilization (Table 3). IAS therefore appears to reflect both baseline immune–inflammatory vulnerability and dynamic disease activity, reinforcing its role as a state-sensitive biomarker rather than a fixed measure of immune aging burden.

**Table 3:** Overall longitudinal changes in immune biomarkers and IAS from admission to remission among patients with complete paired samples (*n* = 83)

Variable	Admission Period	Remission Period	*P*-value
PD-1⁺CD4⁺ T cells (%)	18.2 [13.0–22.6]	13.4 [9.3–17.5]	< 0.001
CD28⁻CD8⁺ T cells (%)	36.7 [29.5–44.1]	31.6 [25.4–39.2]	0.002
IL-6 (pg/mL)	167.1 [134.5–224.7]	58.5 [47.1–78.6]	< 0.001
IAS Score	5.82 ± 1.31	4.67 ± 1.15	< 0.001

Data are presented as median [IQR] or mean ± SD, as appropriate. Paired comparisons were performed using paired t-test for normally distributed variables and Wilcoxon signed-rank test for non-normally distributed variables. Only patients with complete paired admission-remission biomarker data (*n* = 83) were included in longitudinal analyses

### Clinical outcome associations of IAS

A total of 41 deaths occurred within 90 days in the derivation cohort. In the multivariable Cox model including seven predictors (IAS plus six adjustment covariates), the events-per-variable (EPV) ratio was approximately 5.9., indicating limited but acceptable model stability for exploratory prognostic analysis. In the prognostic validation analysis (non-outcome construction), higher IAS (reflecting increased immune–inflammatory vulnerability, IMV) was associated with longer hospital stays and longer antibiotic treatment durations (see Table 4). However, the association with antibiotic treatment duration should be interpreted cautiously, as antibiotic prescribing duration may also be substantially influenced by infection classification, clinician decision-making, and treatment practices rather than IAS alone. Higher IAS was also linked to an increased risk of 30-day readmission and 90-day all-cause mortality (see Table 5).

**Table 4:** Associations Between IAS (Reflecting IMV) and Continuous Clinical Outcomes (Length of Hospital Stay and Antibiotic Treatment Duration)

Outcome	Statistical Method	Effect Size	*P*-value
Length of hospital stay (days)	Spearman correlation	*r* = 0.45	< 0.001
Antibiotic treatment duration (days)	Spearman correlation	*r* = 0.39	0.002

Results represent cross-sectional associations between IAS and continuous clinical outcomes in the derivation cohort. These associations reflect correlation analyses and do not imply causality, nor do they represent paired longitudinal measurements

**Table 5:** Cox proportional hazards regression analysis for 90-day all-cause mortality (41 events; EPV = 5.9)

Variable	HR	95% CI	*P*-value
High IAS (vs. low IAS)	3.42	2.01–5.82	< 0.001
Age (per 1-year increase)	1.03	1.01–1.05	0.004
Male	1.21	0.78–1.89	0.392
GOLD stage (III–IV vs. I–II)	1.76	1.12–2.78	0.015
CRP (per 10 mg/L increase)	1.08	1.02–1.15	0.011
PCT (per 1 ng/mL increase)	1.14	1.03–1.26	0.009
BODE score (per 1 point increase)	1.29	1.11–1.49	< 0.001

With 41 deaths and seven predictors included in the model, the EPV was approximately 5.9. The seven predictors comprised IAS and six adjustment covariates: age, sex, GOLD stage, CRP, PCT, and BODE score

### Predictive performance of IAS compared with conventional clinical scores

To assess the prognostic performance of IAS, we compared its predictive ability with established clinical risk scores, including the BODE (Body mass index, airflow obstruction, dyspnea, exercise capacity) and DECAF (Dyspnea, Eosinophils, Consolidation, Acidemia, Atrial fibrillation) scores.

During the 90-day follow-up period, a total of 41 deaths occurred among the study population. Using the predefined cutoff (Rescaled IAS = 5.30), 96 patients (43.8%) were classified into the high-IAS group and 123 (56.2%) into the low-IAS group in the derivation cohort. Ninety-day mortality was significantly higher in the high-IAS group than in the low-IAS group (31.3% vs. 9.8%, *P* < 0.001). In the primary multivariable Cox regression model, IAS and six additional adjustment covariates were included, yielding an events-per-variable (EPV) ratio of approximately 5.9. Although this EPV was below the conventional threshold for optimal model stability, Firth penalized likelihood estimation was applied to reduce small-sample bias, mitigate overfitting risk, and improve the robustness of coefficient estimates. Corresponding 30-day readmission and 90-day mortality event counts in the external validation cohort are summarized in Supplementary Table S4.

In predicting 90-day all-cause mortality, the IAS demonstrated good discrimination, with an AUC of 0.824 (95% CI: 0.75–0.89), outperforming both the BODE score (AUC = 0.713, 95% CI: 0.632–0.786) and the DECAF score (AUC = 0.741, 95% CI: 0.664–0.803) (both *P* < 0.05).

When IAS was combined with the BODE score, the model’s discriminatory ability further improved (AUC = 0.882, 95% CI: 0.813–0.929), accompanied by significant improvements in net reclassification index (NRI = 0.21) and integrated discrimination improvement (IDI = 0.17). Similarly, combining IAS with the DECAF score increased the AUC to 0.873 (95% CI: 0.806–0.921), indicating superior predictive performance compared to either model alone.

To evaluate whether IAS performance was primarily driven by acute inflammatory signals, we conducted sensitivity analyses comparing the full IAS with simplified models. IL-6 alone yielded an AUC of 0.72, which was lower than that of the full IAS (AUC = 0.824). A reduced model excluding IL-6, incorporating only PD-1⁺CD4⁺ and CD28⁻CD8⁺ T-cell markers, achieved an AUC of 0.79. These findings suggest that although IL-6 contributes substantially to discrimination, IAS captures broader immune-related information beyond a single inflammatory biomarker. This supports the interpretation of IAS as a composite indicator of immune–inflammatory vulnerability, integrating both inflammatory activity and immune senescence-related features, rather than functioning solely as a single-pathway inflammatory or immune aging metric.

It should be noted that the AUC values reported in this section were derived from prognostic models using 90-day all-cause mortality as the endpoint, whereas the discrimination performance of IAS reported in  Immune phenotype classification performance of IAS for distinguishing infection-related versus non-infection-related AECOPD section reflects its ability to distinguish infection-related from non-infection-related AECOPD immune phenotypes. These analyses represent distinct model applications with different biological and clinical objectives; therefore, direct numerical comparison between these AUC values should be interpreted with caution.

Overall, these findings indicate that IAS provides meaningful incremental prognostic value beyond conventional clinical scoring systems by capturing an additional biological dimension related to immune–inflammatory vulnerability. Rather than serving solely as a static immune-aging measure, IAS appears to integrate immune remodeling and dynamic inflammatory responses, thereby enhancing risk stratification for adverse short-term outcomes in older adults with AECOPD.

### Survival analysis and multivariable prognostic validation of IAS

**Fig. 2:**
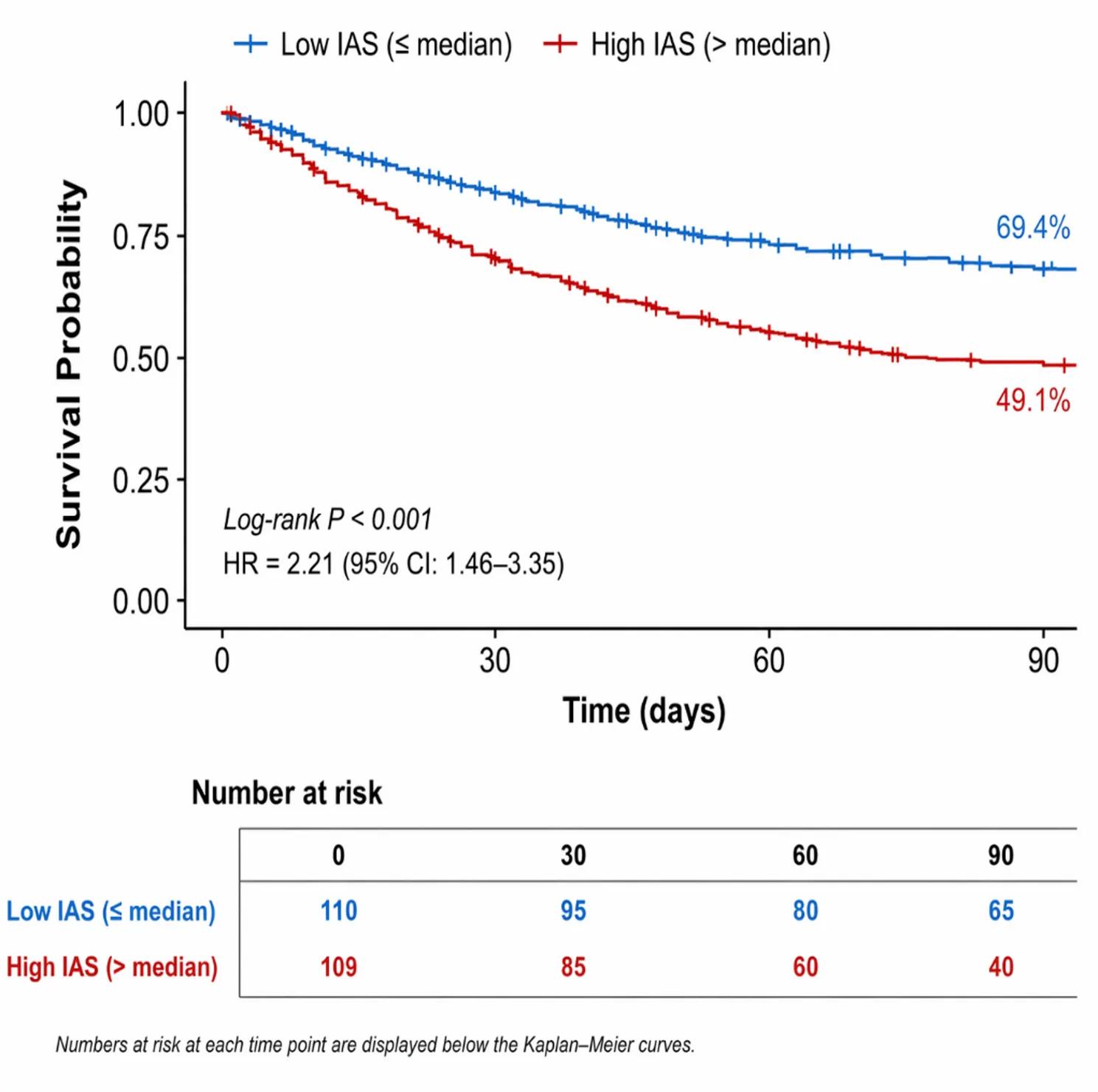
Kaplan–Meier survival curves comparing 90-day all-cause mortality between High IAS (IAS ≥ 5.30) and Low IAS (IAS < 5.30) groups

Kaplan–Meier survival analysis revealed that patients in the high IAS group had significantly higher 90-day all-cause mortality risk compared to those in the low IAS group (log-rank *P* < 0.001) (Fig. 2). In a multivariable Cox proportional hazards model, after adjusting for age, sex, GOLD stage, C-reactive protein (CRP), procalcitonin (PCT), and BODE score, high IAS (reflecting increased IMV) remained significantly associated with an increased 90-day mortality risk (HR = 3.42, 95% CI: 2.01–5.82, *P* < 0.001), indicating that IAS has stable prognostic discrimination ability.

As a sensitivity analysis, we further used a multivariable logistic regression model to evaluate the relationship between IAS and 90-day mortality. The results showed that high IAS (reflecting higher IMV) still significantly increased the 90-day mortality risk (aOR = 3.58, 95% CI: 2.07–6.19, *P* < 0.001), consistent with the Cox model results, suggesting good robustness of the study’s conclusions.

In addition to mortality outcomes, IAS was also significantly associated with multiple clinical adverse outcomes. Compared to the low IAS group, patients in the high IAS group had significantly longer hospital stays and higher 30-day readmission risks (both *P* < 0.05). When IAS was combined with traditional risk assessment tools (e.g., the BODE score), the model’s discriminatory ability further improved, suggesting that IAS can provide incremental predictive value to traditional clinical scoring systems by capturing IMV-related biological vulnerability.

It is important to note that all survival analyses were conducted using Cox proportional hazards models and verified using the Schoenfeld residuals test for the proportional hazards assumption. Kaplan–Meier curves and log-rank tests were used to describe survival differences between IAS groups, while logistic regression was used as a supplementary analysis to verify the robustness of the results.

Patients with high IAS showed significantly reduced survival probability compared with the low IAS group (log-rank test, *P* < 0.001). The shaded areas represent 95% confidence intervals. Hazard ratio (HR) with 95% confidence interval was calculated using Cox proportional hazards regression.

### Exploratory immune–inflammatory phenotype clustering, stability analysis, and clinical characterization

Exploratory unsupervised clustering based on key immune–inflammatory markers associated with immune–inflammatory vulnerability (IMV) identified three major immune–inflammatory profiles among all AECOPD patients. Clinical characteristics across these clusters are summarized in Table 6. Significant heterogeneity was observed across multiple demographic, inflammatory, and prognostic parameters. Cluster A demonstrated features suggestive of relatively lower inflammatory activity and lower immune exhaustion, Cluster B showed an inflammation-dominant pattern, and Cluster C was characterized by combined inflammatory activation and higher clinical risk. Patients in Cluster C tended to be older, had a higher prevalence of comorbidities such as hypertension and diabetes, showed elevated IL-6 and NLR levels, and experienced longer hospital stays, prolonged antibiotic treatment durations, and higher 30-day readmission and 90-day mortality rates than those in Clusters A and B (all *P* < 0.05). Importantly, these exploratory clusters reflect broader multidimensional immune heterogeneity and should not be interpreted as direct confirmation of the comparatively higher exhaustion–lower inflammation pattern observed in supervised infection-stratified analyses. Instead, they indicate trends suggestive of possible immune–inflammatory vulnerability states in this cohort.

**Table 6:** Comparison of clinical characteristics across immune phenotype groups

Variable	Cluster A (*n* = 75)	Cluster B (*n* = 64)	Cluster C (*n* = 80)	*P*-value
Age (years)	68.3 ± 7.4	70.1 ± 6.5	73.6 ± 5.9	0.021
Male (%)	62.5%	66.7%	70.8%	0.741
Hypertension (%)	34.4%	46.7%	62.5%	0.037
Diabetes (%)	18.8%	30.0%	41.7%	0.042
Hospital stay (days)	6.8 ± 2.1	8.9 ± 2.7	11.2 ± 3.4	< 0.001
Antibiotic use duration (days)	4.1 ± 1.3	6.2 ± 2.0	7.5 ± 2.3	< 0.001
30-day readmission (%)	6.3%	13.3%	25.0%	0.008
90-day mortality (%)	0.0%	10.7%	30.6%	0.001

This table presents cross-sectional comparisons across immune phenotype groups and does not represent paired longitudinal measurements

To further evaluate clustering robustness, consensus clustering with 1,000 resampling iterations and silhouette coefficient analyses were performed. The three-cluster solution (k = 3) demonstrated clear separation in consensus matrices and achieved an average silhouette score of 0.72, indicating stable internal consistency and reliable clustering structure. Patients in Cluster C exhibited the highest 90-day mortality risk (26.3%), consistent with the adverse prognostic trend observed in the high-IAS group. These findings support the reproducibility and potential biological plausibility of the identified immune–inflammatory phenotypes, while acknowledging that the observations are specific to this cohort and should be interpreted cautiously.

### Immune–inflammatory marker differences in clinical subgroups

Among the 98 patients in the non-infection-related AECOPD group, 83 had complete immune phenotyping, metabolic syndrome classification, and environmental exposure data and were therefore included in subgroup analyses. The remaining 15 patients were excluded due to incomplete subgroup-defining data, precluding reliable phenotype classification. In the non-infection-related AECOPD group, patients with metabolic syndrome (MetS) had significantly higher PD-1⁺CD4⁺ T cell levels compared to those without MetS (21.8% [17.1–26.4] vs. 16.2% [12.6–19.4], *P* = 0.008). There was also a trend towards increased CD28⁻CD8⁺ T cells (*P* = 0.067). Additionally, individuals with a history of environmental exposure (*n* = 28) showed slightly higher PD-1 expression, though this did not reach statistical significance (*P* = 0.092). These results suggest a potential link between chronic metabolic inflammation and immune–inflammatory alterations associated with IMV, which may play an important role in shaping the immune phenotype in non-infection-related AECOPD (see Table 7).

**Table 7:** Comparison of Immune–inflammatory Markers Across Clinical Subgroups in Non-infection-related AECOPD Patients (Median [IQR])

Variable	Metabolic Syndrome Group (*n* = 34)	Non-MetS Group (*n* = 49)	*P*-value	Environmental Exposure Group (*n* = 28)	Non-exposure Group (*n* = 55)	*P*-value
PD-1⁺CD4⁺ T cells (%)	21.8 [17.1–26.4]	16.2 [12.6–19.4]	0.008	19.6 [15.2–24.1]	17.2 [13.4–21.6]	0.092
CD28⁻CD8⁺ T cells (%)	37.4 [31.3–42.5]	34.1 [27.8–39.7]	0.067	36.1 [30.2–40.9]	35.2 [28.1–39.4]	0.284
mHLA-DR Expression (MFI)	8,417 [6,932–9,603]	9,012 [7,482–10,227]	0.173	8,654 [7,132–9,774]	9,106 [7,402–10,184]	0.289
IL-6 (pg/mL)	112.6 [88.4–137.9]	97.1 [72.5–124.3]	0.044	108.3 [83.7–130.5]	96.4 [75.2–122.9]	0.065
IAS Score	5.43 ± 1.26	4.87 ± 1.18	0.023	5.36 ± 1.19	5.02 ± 1.21	0.148

Subgroup analysis was performed among non-infection-related AECOPD patients with complete data on environmental exposure. A total of 15 patients were excluded due to missing exposure information, resulting in 83 patients included in the analysis

mHLA-DR data were available only in a subset of patients and were therefore analyzed in subgroup analyses

## Discussion

This study proposes a biomarker-based risk stratification framework reflecting IMV in older adults with AECOPD. Rather than focusing solely on inflammatory intensity, IAS integrates T-cell exhaustion, terminal differentiation, and systemic inflammation, thereby capturing both aging-related immune remodeling and dynamic inflammatory responses. In geriatric populations, where physiological reserve and adaptive capacity decline, such multidimensional immune profiling may better characterize the heterogeneity of exacerbation mechanisms and clinical outcomes compared with traditional inflammation-centered approaches.

### Immune–inflammatory phenotypes in infection-related AECOPD

In the present study, infection-related AECOPD demonstrated higher absolute levels of acute inflammatory and immune activation markers at admission, including IL-6, PD-1⁺CD4⁺ T cells, and CD28⁻CD8⁺ T cells, indicating that acute infection is associated with a biologically intense state characterized by concurrent systemic inflammation and immune activation/exhaustion. These findings suggest that infection-triggered exacerbations are not solely inflammatory events, but may also involve substantial immune remodeling, reflecting the complex interaction between pathogen burden, host inflammatory response, and aging-related immune dysfunction. Elevated IL-6 in this context likely reflects acute inflammatory amplification, whereas increased PD-1⁺CD4⁺ and CD28⁻CD8⁺ T-cell proportions may indicate superimposed immune exhaustion or accelerated senescence under inflammatory stress.

Importantly, although non-infection-related AECOPD was comparatively associated with lower overt inflammatory burden relative to infection-related cases in the present analytical framework, this should not be interpreted as a biologically quiescent or immunologically normal state. Lower apparent inflammatory intensity does not necessarily equate to lower biological vulnerability, as chronic oxidative stress, environmental exposures, metabolic dysregulation, cardiovascular burden, and baseline inflammaging may contribute to ongoing immune–inflammatory vulnerability even in the absence of overt infection.

These observations reinforce that AECOPD represents a heterogeneous immune–inflammatory spectrum rather than a simple binary distinction between infectious and non-infectious triggers. While infection-related AECOPD may represent the highest absolute inflammatory and immune activation state at presentation, non-infection-related AECOPD may still encompass biologically vulnerable states not fully captured by conventional inflammatory markers alone. This framework better aligns with contemporary multidimensional COPD heterogeneity models and supports the broader concept that immune phenotype stratification should extend beyond trigger classification alone.

Accordingly, IAS should primarily be interpreted as a biologically informed IMV framework integrating dynamic inflammatory responsiveness with aging-related immune remodeling, rather than as a direct infection classifier or a static measure of immutable immune-aging burden. This interpretation better reflects the observed biomarker distributions, explains the partial remission-associated decline in IAS, and provides a more conceptually consistent foundation for understanding biological vulnerability across heterogeneous AECOPD states.

### Immune–inflammatory mechanisms in non-infection-related AECOPD: metabolic stress, terminal differentiation, and clinical features

Within the broader IMV framework described above, this section specifically focuses on phenotype heterogeneity in non-infection-related AECOPD rather than on the broader prognostic role of IAS.

In contrast to infection-related AECOPD, patients with non-infection-related exacerbations showed adjusted biomarker associations suggestive of comparatively greater T-cell exhaustion/terminal differentiation features under lower overt inflammatory conditions. These findings, observed in this cohort, may reflect IMV and metabolic-stress-related features, but should be interpreted cautiously and do not define a fixed immune phenotype.

Patients with metabolic syndrome (MetS) exhibited higher levels of PD-1⁺CD4⁺ T cells and elevated IAS scores, indicating that metabolic abnormalities may contribute to immune exhaustion. Chronic metabolic stress, driven by mitochondrial dysfunction and altered nutrient sensing pathways, can induce T cell exhaustion even in the absence of significant inflammation, reinforcing persistent maladaptive immune remodeling and biological vulnerability [27, 28].

Interestingly, although inflammation markers such as PCT did not reach the infection threshold, IL-6 levels remained elevated, suggesting a chronic, low-grade inflammatory state consistent with the concept of “inflammaging,” where persistent, low-level inflammation coexists with immune dysfunction and metabolic dysregulation [29, 30]. This diminished adaptive capacity to metabolic load, environmental stressors, or comorbidities leads to immune imbalance and exacerbation without a typical acute inflammatory response. These immune exhaustion patterns have been observed in sepsis and other critical conditions, though the specific mechanisms and disease contexts differ [21]. Thus, AECOPD may share overlapping IMV pathways with other conditions while retaining disease-specific characteristics.

While mechanisms such as T cell exhaustion and metabolic stress may be mediated by pathways including mTOR/AMPK signaling and mitochondrial dysfunction, this study primarily provides observational evidence. Further experimental validation is required to clarify their roles in shaping IMV in AECOPD patients. Collectively, these findings support that biologically relevant non-infectious AECOPD heterogeneity may extend beyond trigger-based classification alone and may reflect broader interactions among metabolic stress, inflammaging, and immune maladaptation.

### Clinical value of IAS and incremental risk stratification

Building on the phenotype-specific biological distinctions described above, this section focuses specifically on whether IAS provides independent prognostic information for short-term adverse outcomes rather than readdressing its mechanistic basis.

IAS may provide a biologically informed framework for short-term biological risk stratification in older adults with AECOPD, rather than an immediately implementable stand-alone clinical decision-making tool. IAS should be interpreted not simply as a conventional inflammatory index but as a composite biomarker reflecting IMV in this population. Although IAS was retained as the established abbreviated name of the index, it is interpreted in the present study as an immune–inflammatory vulnerability index rather than a direct measure of fixed immune-aging burden. Importantly, IAS was designed as a biomarker-based composite framework to capture IMV-related heterogeneity across AECOPD states rather than to function as a direct infection classifier. This conceptual framework suggests that IAS may offer incremental biological stratification beyond conventional inflammatory markers; however, the present observational study does not establish that IAS-guided management changes clinical decisions or improves patient outcomes. Accordingly, IAS should currently be viewed primarily as a research-supported prognostic stratification framework, while the precise mechanistic pathways linking IMV to adverse outcomes remain incompletely understood and warrant further prospective and experimental investigation.

Sensitivity analyses further support that IAS is not solely a surrogate for acute inflammatory burden. Although IL-6 was markedly elevated in infection-related AECOPD, IL-6 alone demonstrated lower discriminatory performance than the full IAS. Moreover, a reduced model excluding IL-6 retained meaningful performance, suggesting that T-cell exhaustion (PD-1⁺CD4⁺) and senescence/terminal differentiation (CD28⁻CD8⁺) may contribute independently to IMV characterization. These findings further support IAS as a multidimensional biomarker-based stratification framework that extends beyond single-pathway inflammatory burden alone. Nevertheless, broader translational application will require prospective validation, multicenter assay harmonization, feasibility assessment across laboratory settings, and demonstration of clinical utility before routine implementation can be considered.

#### Discriminatory ability compared to traditional scoring systems

IAS (reflecting IMV) demonstrated robust discriminatory ability in predicting 90-day all-cause mortality. When integrated with established multidimensional clinical frameworks such as BODE and DECAF, overall model discrimination improved further, suggesting that IAS may provide incremental prognostic information beyond conventional clinical scoring systems alone. Importantly, these findings support IAS primarily as a complementary biomarker-based stratification tool rather than a replacement for established prognostic models.

#### Reclassification improvement

When integrated with established multidimensional clinical frameworks such as BODE and DECAF, overall model discrimination improved further, suggesting that IAS may provide incremental prognostic information beyond conventional clinical scoring systems alone. Importantly, these findings support IAS primarily as a complementary biomarker-based stratification tool rather than a replacement for established prognostic models.

#### Clinical utility and net benefit

Because improved statistical discrimination does not inherently ensure clinical usefulness, decision curve analysis (DCA) was performed to evaluate potential net clinical benefit across threshold probabilities. Results showed that, within a reasonable threshold probability range, the model incorporating IAS achieved higher net benefit, suggesting that IAS-informed models may improve identification of biologically vulnerable higher-risk patients under equivalent resource conditions. However, these findings should be interpreted as evidence of potential stratification utility rather than proof that IAS-directed intervention strategies independently improve outcomes. At present, IAS may be most appropriately viewed as a framework for enhancing short-term biological risk recognition and prioritization rather than as a stand-alone therapeutic decision instrument.

#### Implementable application scenarios and suggested process

From an operational and translational perspective, IAS incorporates a limited number of quantifiable biomarkers, which may support early-stage biological risk stratification in older adults with AECOPD at hospital admission, particularly in settings with standardized immune phenotyping capacity. Importantly, IAS is intended to complement rather than replace established clinical scoring systems. A pragmatic stepwise framework may therefore be considered for future implementation, although this approach remains conceptual and requires prospective validation. Initial risk assessment can be performed using conventional tools (e.g., BODE or DECAF scores) combined with routine laboratory tests. IAS may then be calculated in parallel to provide an additional dimension reflecting IMV. For patients with intermediate risk or discordant clinical findings, integrating IAS with traditional scoring systems may further refine risk stratification. Patients identified as biologically higher risk through this combined framework may represent candidates for closer monitoring, more frequent reassessment, or structured follow-up prioritization; however, the present study does not establish that IAS-guided interventions independently improve outcomes or should directly alter treatment decisions.

It should be noted that cardiopulmonary and circulatory interactions, including myocardial injury, cardiac overload, and hemodynamic alterations, may influence clinical outcomes in AECOPD and potentially interact with immune and hematopoietic function [11, 21]. Evidence from cardiovascular research further suggests that biomarkers of myocardial injury may have prognostic value in critically ill patients [31]. However, cardiovascular parameters such as Troponin T, NT-proBNP, and LVEF were not included in the present study; therefore, The incomplete sentence and citation formatting in this paragraph were corrected to ensure grammatical completeness and proper integration of the supporting reference(s).

### Clinical translation potential: from phenotype recognition to individualized interventions

This study suggests that IAS may serve as a biologically informed adjunctive framework for identifying older adults with AECOPD who exhibit distinct immune-inflammatory profiles and varying short-term prognostic vulnerability. Unlike traditional assessments based primarily on symptoms and physiological parameters, IAS provides a biologically informed dimension by capturing underlying IMV.

When integrated with established multidimensional risk scores (e.g., BODE, DECAF), IAS demonstrated incremental prognostic stratification capacity in the present cohort. This indicates that IMV-related information may provide additional biological and prognostic context beyond conventional clinical indicators. However, these findings should not be interpreted as evidence that IAS-guided management independently improves outcomes, and substantial practical barriers currently limit broad implementation.

The IAS model relies on flow cytometry-based measurement of PD-1⁺CD4⁺ and CD28⁻CD8⁺ T cells, which requires specialized equipment, standardized antibody panels, and trained personnel. Consequently, it may not be readily available in primary care or resource-limited settings. Laboratory variability, including differences in gating strategies, instruments, and reagents, can affect measurement consistency and cross-center comparability. Compared with routine laboratory testing, immune phenotyping is more resource-intensive and time-consuming, which may reduce applicability in time-sensitive or infrastructure-limited scenarios. Accordingly, IAS should currently be regarded primarily as an adjunctive biomarker-based stratification framework, most appropriate for tertiary care, centralized laboratory networks, or research settings with standardized immunological infrastructure, rather than as a universal frontline screening instrument.To enhance accessibility and translational feasibility, future investigations should prioritize development and validation of simplified, surrogate, or computationally assisted models—potentially incorporating routine laboratory parameters, centralized testing pipelines, or machine learning approaches—while preserving core IMV-related biological relevance. These strategies may facilitate broader implementation across diverse healthcare environments.

From a geriatric and precision-risk perspective, IAS appears most valuable as a complementary rather than replacement framework. In older adults with discordant clinical, inflammatory, or functional findings, IAS may provide additional insight into underlying IMV-related biological vulnerability, potentially informing monitoring intensity, reassessment prioritization, or follow-up planning. Overall, IAS may add a meaningful biological dimension to multidimensional risk evaluation by incorporating IMV into vulnerability assessment, although prospective interventional validation remains necessary before individualized IAS-guided management strategies can be recommended.

### Limitations and future directions

Despite its strengths in study design and internal consistency, this study has several limitations.

First, this was a single-center cohort study with a relatively small number of 90-day mortality events. The relatively limited number of events resulted in an events-per-variable (EPV) ratio below the conventional threshold, which may increase the risk of model overfitting. Although variable selection strategies and the Firth penalized likelihood method were applied to mitigate small-sample bias, these findings should be interpreted with caution, and external validation in larger, multi-center cohorts is required.

Second, potential misclassification in distinguishing infection-related from non-infection-related AECOPD should be acknowledged. Although we used a prespecified hierarchical adjudication protocol incorporating microbiological evidence, biomarker thresholds, clinical manifestations, and blinded specialist review, infection classification in AECOPD remains inherently complex. To minimize incorporation bias, treatment response was not used as a primary classification criterion, and immune phenotyping results were unavailable to adjudicators. In this cohort, 61.2% of infection-related cases were microbiologically confirmed, only 13.2% required specialist adjudication, and inter-rater agreement for uncertain cases was substantial (Cohen’s κ = 0.82). Moreover, sensitivity analyses using stricter infection definitions yielded consistent findings, supporting the robustness of IAS despite classification complexity. Nevertheless, future multicenter studies with more standardized microbiological and molecular diagnostic protocols are needed.

Third, cardiac biomarkers and cardiovascular functional parameters (e.g., Troponin T, NT-proBNP, and LVEF) were not assessed. Given the well-recognized cardiopulmonary and circulatory interactions in AECOPD, their absence may introduce residual confounding and limit comprehensive prognostic evaluation. Potential associations between cardiovascular function and IAS or other immune–inflammatory markers could therefore not be explored. Accordingly, the independent contribution of IMV to clinical outcomes should be interpreted with caution. Future studies incorporating cardiac biomarkers and functional parameters are needed to clarify the interplay between IMV, circulatory dysfunction, and multi-organ interactions.

Fourth, several practical and methodological limitations may affect IAS implementation. The current model relies on flow cytometry-based measurement of PD-1⁺CD4⁺ and CD28⁻CD8⁺ T cells, which requires specialized equipment, standardized antibody panels, and trained personnel. As a result, it may not be feasible in primary care or resource-limited settings.

In addition, variability across laboratories may influence measurement consistency. Differences in gating strategies, instrument platforms, and reagent selection may reduce reproducibility and limit cross-center comparability. Although all assays in this study were conducted under standardized conditions, real-world variability may affect IAS robustness.

Cost and turnaround time also represent important constraints. Compared with routine laboratory tests, immune phenotyping is more resource-intensive and time-consuming, which may reduce applicability in time-sensitive clinical scenarios.

Another limitation is that the association between IAS dynamics and clinical outcomes was not formally assessed. Although changes in IAS were observed, the prognostic significance of these remission-associated dynamic changes was not a prespecified objective, and their relationship with specific clinical outcomes therefore remains uncertain and requires further evaluation in future longitudinal studies.

Finally, while IAS demonstrated good predictive performance, its current structure may limit scalability. Future studies should focus on developing simplified or surrogate models, based on routinely available laboratory parameters or computational approaches such as machine learning. These strategies may improve accessibility while preserving clinical relevance.

Overall, despite these limitations, this study provides a biologically grounded framework for understanding the heterogeneity of IMV in AECOPD and establishes an initial foundation for future multicenter validation, mechanistic investigation, and prospective translational research. These findings support IAS as a biologically informed, research-oriented indicator of IMV in older adults; however, broader clinical applicability will require further validation of reproducibility, feasibility, and clinical utility before routine implementation can be considered.

## Conclusion

This study developed and externally validated an IMV oriented score, IAS, based on PD-1⁺CD4⁺ T cells, CD28⁻CD8⁺ T cells, and IL-6. Within the present supervised biological framework, IAS demonstrated discriminatory capacity for differentiating infection-related from non-infection-related AECOPD and showed prognostic relevance for short-term adverse clinical outcomes, with stable performance in an independent external cohort.

These findings support the biological and clinical relevance of IMV as a potentially important dimension associated with immune–inflammatory heterogeneity and short-term outcomes in AECOPD.

In supervised comparative analyses, non-infection-related exacerbations were comparatively associated with relatively greater immune exhaustion under lower inflammatory conditions; however, this adjusted association pattern should be interpreted within the context of the present study framework rather than as a universal immune phenotype or definitive biological subtype.

Rather than representing a fixed immune-aging burden, IAS reflects a composite biomarker-based dimension of IMV that integrates dynamic inflammatory and immune features. Accordingly, IAS may serve as a biologically informed, research-oriented framework for future risk stratification investigation in older adults with AECOPD. However, prospective multicenter validation, feasibility assessment, and demonstration of real-world clinical utility remain necessary before IAS-guided management or routine clinical implementation can be recommended.

## Supplementary Information

Supplementary Material 1.

Supplementary Material 2.

Supplementary Material 3.

## Data Availability

The datasets generated and/or analyzed during the current study are not publicly available due to institutional restrictions but are available from the corresponding author on reasonable request.
