# Supplementary material for: Immune–inflammatory vulnerability index for risk stratification in older adults with acute exacerbations of COPD: a prospective cohort study
Source: BMC Geriatr. 2026 Jun 29;26:1072. doi: 10.1186/s12877-026-07863-3 (PMC13495256; doi:10.1186/s12877-026-07863-3)
Supplement: Supplementary file 1 [file 12877_2026_7863_MOESM1_ESM.docx]

**Supplementary**

**Supplementary Figure S1. Calibration of the immune aging score (IAS)**

**Supplementary Figure S1.** Calibration performance of the immune aging score (IAS) in the training and external validation cohorts.
**(A)** Calibration curve in the training cohort, showing the agreement between predicted and observed event probabilities. The dotted diagonal line represents ideal calibration. Circles indicate apparent performance across deciles of predicted risk, and the dashed line represents the bootstrap-corrected calibration curve based on 1,000 resamples.
**(B)** Calibration curve in the external validation cohort. Observed outcome frequencies across deciles of predicted risk are plotted against predicted probabilities, with the smoothed curve illustrating overall calibration performance.

**Supplementary Figure S2. Decision curve analysis (DCA) of the immune aging score**

**Supplementary Figure S2.** Decision curve analysis (DCA) comparing the clinical net benefit of the immune aging score (IAS) with conventional risk scores in the training and external validation cohorts.
**(A)** DCA in the training cohort.
**(B)** DCA in the external validation cohort.

Across a wide range of threshold probabilities (0.10–0.80), IAS consistently demonstrates a higher net benefit compared with the “treat-all” and “treat-none” strategies, as well as established clinical scores (BODE and DECAF), indicating superior clinical utility for risk stratification and decision-making.
