# Supplementary material for: Immune–inflammatory vulnerability index for risk stratification in older adults with acute exacerbations of COPD: a prospective cohort study
Source: BMC Geriatr. 2026 Jun 29;26:1072. doi: 10.1186/s12877-026-07863-3 (PMC13495256; doi:10.1186/s12877-026-07863-3)
Supplement: Supplementary file 2 [file 12877_2026_7863_MOESM2_ESM.docx]

**Supplementary Materials Contents**

**Supplementary Table S1. STROBE Checklist for Reporting Observational Studies**
**Supplementary Table S2. Candidate Variables and Final LASSO Coefficients for IAS Construction**
**Supplementary Table S3. Baseline Characteristics of the Derivation and External Validation Cohorts**
**Supplementary Table S4. Clinical Outcome Event Counts in the Derivation and External Validation Cohorts**

**Supplementary Table S1. STROBE Checklist**

| **Section/Item** | **Item No.** | **Recommendation** | **Reported on Page No.** |
| --- | --- | --- | --- |
| **Title and Abstract** | 1 | Indicate the study’s design with a commonly used term in the title or the abstract | Title; Abstract |
|  | 2 | Provide in the abstract an informative and balanced summary of what was done and what was found | Abstract |
| **Introduction** | 3 | Explain the scientific background and rationale for the investigation being reported | Introduction |
|  | 4 | State specific objectives, including any prespecified hypotheses | Introduction |
| **Methods** | 5 | Present key elements of study design early in the paper | Section 2.1 |
|  | 6 | Describe the setting, locations, and relevant dates, including periods of recruitment, exposure, and follow-up | Section 2.1 |
|  | 7 | Give the eligibility criteria, and the sources and methods of selection of participants | Section 2.1 |
|  | 8 | Clearly define all outcomes, exposures, predictors, potential confounders, and effect modifiers | Sections 2.2–2.4 |
|  | 9 | Describe sources of data and details of methods of assessment (measurement) | Section 2.3 |
|  | 10 | Describe any efforts to address potential sources of bias | Sections 2.1, 2.2, 2.5 |
|  | 11 | Explain how the study size was arrived at | Section 2.1 |
|  | 12 | Explain how quantitative variables were handled in the analyses | Sections 2.4–2.7 |
|  | 13 | Describe all statistical methods, including those used to control for confounding | Sections 2.5–2.7 |
| **Results** | 14 | Report numbers of individuals at each stage of study (e.g., potentially eligible, examined for eligibility, included, completed follow-up, and analyzed) | Section 3.1; Figure 1 |
|  | 15 | Describe characteristics of study participants | Section 3.1; Table 1 |
|  | 16 | Report outcome events or summary measures | Sections 3.2–3.5; Tables 3–4 |
|  | 17 | Report other analyses (e.g., subgroup analyses, sensitivity analyses, external validation analyses) | Sections 3.2–3.5; Supplementary Tables/Figures |
| **Discussion** | 18 | Summarize key results with reference to study objectives | Discussion |
|  | 19 | Discuss limitations of the study, taking into account sources of potential bias or imprecision | Section 4.5 |
|  | 20 | Give a cautious overall interpretation of results considering objectives, limitations, multiplicity of analyses, and relevant evidence | Discussion; Conclusion |
|  | 21 | Discuss the generalizability (external validity) of the study results | Sections 4.4–4.5 |
| **Other Information** | 22 | Give the source of funding and the role of the funders for the present study | Funding Statement / Declarations |

**Abbreviation:** STROBE, Strengthening the Reporting of Observational Studies in Epidemiology.

**Note:** A participant flow diagram detailing screening, enrollment, exclusions, derivation cohort inclusion, paired-sampling completion, follow-up completeness, and external validation cohort selection is provided in Figure 1.
**Note:** This checklist is provided in accordance with BMC Geriatrics reporting requirements for observational studies.

**Supplementary Table S2. Candidate Variables and Final LASSO Coefficients for IAS Construction**

| **Candidate variable** | **Biological domain** | **Transformation** | **Final coefficient** |
| --- | --- | --- | --- |
| PD-1⁺CD4⁺ T cells | T-cell exhaustion | Z-score | 0.38 |
| CD28⁻CD8⁺ T cells | T-cell terminal differentiation/senescence | Z-score | 0.33 |
| IL-6 | Systemic inflammation / inflammaging | log10 transformation + Z-score | 0.47 |

**Note: Only these three biologically prespecified biomarkers were entered into the LASSO model. No clinical variables, CRP, PCT, or outcome variables were included. Coefficients were not refitted after selection. The raw IAS was calculated as a weighted sum of standardized biomarkers: Raw IAS = 0.38 × Z(PD-1⁺CD4⁺ T cells) + 0.33 × Z(CD28⁻CD8⁺ T cells) + 0.47 × Z(log₁₀IL-6). For clinical interpretability and to avoid negative values, the raw IAS was linearly rescaled to a 0–10 scale using min–max normalization based on the derivation cohort. The high-versus-low IAS threshold of 5.30 was determined from the rescaled IAS using ROC analysis for 90-day all-cause mortality with the Youden index and was applied unchanged to all subsequent analyses, including external validation.**

Supplementary Table S3. **Baseline Characteristics of the Derivation and External Validation Cohorts**

| **Variable** | **Derivation cohort (n=219)** | **Validation cohort (n=136)** | **P value** |
| --- | --- | --- | --- |
| Age (years), mean ± SD | 72.7 ± 6.2 | 73.4 ± 7.1 | 0.344 |
| Male, n (%) | 150 (68.5) | 89 (65.4) | 0.551 |
| Smoking history, n (%) | 126 (57.5) | 75 (55.1) | 0.659 |
| Chronic heart failure comorbidity, n (%) | 73 (33.3) | 36 (26.5) | 0.173 |
| CRP (mg/L), median (IQR) | 56.7 (32.6–78.9) | 61.2 (34.6–80.3) | 0.541 |
| PCT (ng/mL), median (IQR) | 5.62 (3.81–6.35) | 5.01 (3.65–5.90) | 0.425 |
| IL-6 (pg/mL), median (IQR) | 127.6 (98.5–172.5) | 130.1 (86.7–181.2) | 0.678 |
| CD28⁻CD8⁺ T cells (%), mean ± SD | 35.7 ± 11.8 | 37.6 ± 10.1 | 0.108 |
| PD-1⁺CD4⁺ T cells (%), mean ± SD | 16.0 ± 5.6 | 15.2 ± 6.0 | 0.211 |
| CD4⁺/CD8⁺ ratio, mean ± SD | 1.43 ± 0.56 | 1.40 ± 0.51 | 0.604 |
| NLR, mean ± SD | 4.74 ± 2.26 | 4.81 ± 2.32 | 0.780 |
| Length of hospital stay (days), mean ± SD | 10.4 ± 4.1 | 10.3 ± 4.4 | 0.831 |

Continuous variables are presented as mean ± standard deviation (SD) or median (interquartile range, IQR), as appropriate. Categorical variables are presented as number (percentage). Comparisons between groups were performed using Student’s t-test or Mann–Whitney U test for continuous variables and χ² test for categorical variables.

**Supplementary Table S4. Clinical Outcome Event Counts in the Derivation and External Validation Cohorts**

| **Outcome** | **Derivation cohort (n = 219)** | **External validation cohort (n = 136)** |
| --- | --- | --- |
| 30-day readmission, n (%) | 34 (15.5%) | **21 (15.4%)** |
| 90-day all-cause mortality, n (%) | **41 (18.7%)** | **24 (17.6%)** |

**Note:** Clinical outcome event counts are reported to improve transparency regarding prognostic model stability, external validation performance, and events-per-variable considerations. Outcome rates in the external validation cohort were comparable to those in the derivation cohort, supporting the generalizability of IAS performance across independent populations. Mortality and readmission events in the derivation cohort formed the basis for prognostic analyses, including Cox proportional hazards modeling and discrimination analyses.

**Supplementary Figure S1. Calibration performance of the immune aging score (IAS) in the derivation and external validation cohorts.**

**(A)** Calibration curve in the training cohort, showing the agreement between predicted and observed event probabilities. The dotted diagonal line represents ideal calibration. Circles indicate apparent performance across deciles of predicted risk, and the dashed line represents the bootstrap-corrected calibration curve based on 1,000 resamples.
**(B)** Calibration curve in the external validation cohort. Observed outcome frequencies across deciles of predicted risk are plotted against predicted probabilities, with the smoothed curve illustrating overall calibration performance.

**Supplementary Figure S2.** **Decision curve analysis (DCA) comparing the clinical net benefit of the immune aging score (IAS) with conventional risk scores in the training and external validation cohorts.
(A)** DCA in the training cohort.
**(B)** DCA in the external validation cohort.

Across a wide range of threshold probabilities (0.10–0.80), IAS consistently demonstrates a higher net benefit compared with the “treat-all” and “treat-none” strategies, as well as established clinical scores (BODE and DECAF), indicating superior clinical utility for risk stratification and decision-making.
